# Emerging roles for lipids in non-apoptotic cell death

**DOI:** 10.1038/cdd.2016.25

**Published:** 2016-03-11

**Authors:** L Magtanong, P J Ko, S J Dixon

**Affiliations:** 1 Department of Biology, Stanford University, 337 Campus Drive, Stanford, CA, USA

## Abstract

Non-apoptotic regulated cell death (RCD) is essential to maintain organismal homeostasis and may be aberrantly activated during certain pathological states. Lipids are emerging as key components of several non-apoptotic RCD pathways. For example, a direct interaction between membrane phospholipids and the pore-forming protein mixed lineage kinase domain-like (MLKL) is needed for the execution of necroptosis, while the oxidative destruction of membrane polyunsaturated fatty acids (PUFAs), following the inactivation of glutathione peroxidase 4 (GPX4), is a requisite gateway to ferroptosis. Here, we review the roles of lipids in the initiation and execution of these and other forms of non-apoptotic cell death. We also consider new technologies that are allowing for the roles of lipids and lipid metabolism in RCD to be probed in increasingly sophisticated ways. In certain cases, this new knowledge may enable the development of therapies that target lipids and lipid metabolic processes to enhance or suppress specific non-apoptotic RCD pathways.

## Facts

- Emerging evidence suggests important roles for lipids and lipid metabolism in several non-apoptotic cell death pathways.
- Non-apoptotic cell death can be triggered by specific fatty acids.
- Specific lipids in the plasma membrane are essential for the execution of non-apoptotic cell death.
- Depletion of specific lipids is required for at least one form of non-apoptotic RCD.

## Open Questions

- What molecular mechanisms link the accumulation of specific lipids to the induction of non-apoptotic RCD?
- Do lipids or the disruption of lipid metabolic pathways trigger non-apoptotic RCD pathways in unusual ways, or perhaps cause new types of non-apoptotic RCD?
- Can diseases of lipid metabolism teach us anything about how lipids trigger or mediate non-apoptotic cell death?
- Can we specifically target lipid-dependent aspects of non-apoptotic RCD to treat disease?

Regulated cell death (RCD) is crucial for development and the maintenance of homeostasis.^1, 2^ In addition to apoptosis, in recent years many non-apoptotic RCD pathways have been described, including necroptosis, pyroptosis, parthanatos, ferroptosis and several others.^2, 3, 4, 5, 6, 7^ These pathways are known or thought to contribute to cell death following viral infection, bacterial infection, neurodegeneration, ischemia-reperfusion injury to various tissues and other pathological processes, and therefore present new targets for therapeutic intervention.^8, 9^ Understanding the regulation of non-apoptotic RCD pathways is therefore of great biomedical interest.

**Figure 1:**
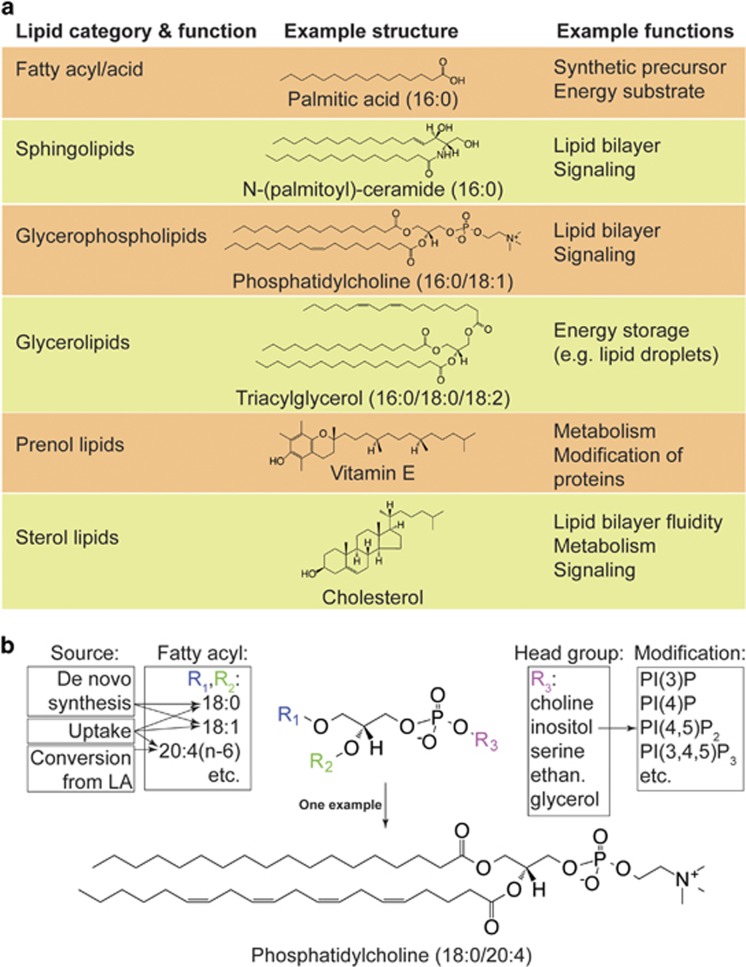
Overview of lipids and lipid diversity. (**a**) Six categories of lipids important for mammalian cell function (see also lipidmaps.org for more information). (**b**) An example of structural diversity in the glycerophospholipid class. Glycerophospholipids can be esterified at two positions (R_1_ and R_2_, respectively) with distinct FAs. SFAs and MUFAs can be synthesized *de novo* or taken up from the environment. PUFAs are taken up from the environment or synthesized from essential PUFA precursors like linoleic acid (LA, 18:2n-6). The head group conjugated to the phosphate can be one of the several molecules (ethan.: ethanolamine). Inositol can be further modified by phosphorylation, generating additional diversity. The example molecule shows a PC conjugated to an SFA, stearic acid (R_1_=18:0), and a PUFA, arachidonic acid (R_2_=20:4n-6)

Lipids and lipid metabolism are emerging as key regulators of cell survival,^10^ proliferation,^11^ stress responses,^12^ and as described in this review, cell death. Six categories of lipids, each with distinguishing structural features, are normally present in mammalian cells: fatty acids (FAs), sphingolipids, glycerolipids, glycerophospholipids, prenol lipids, and sterol lipids (Figure 1a).^13^ Each category of lipids encompasses diverse molecular species. For example, glycerophospholipids, a major constituent of biological membranes, can be subdivided into those containing choline (phosphatidylcholine, PC), inositol (phosphatidylinositol, PI), serine (phosphatidylserine, PS), and other head groups, some of which can be further modified (e.g., phosphorylation of PI to generate phosphatidylinositol phosphates (PIPs))(Figure 1b). Furthermore, each glycerophospholipid can contain esterified FAs with different chain lengths and degrees of unsaturation (corresponding to the number of double bonds in the FA chain), ultimately generating incredible structural diversity (Figure 1b). Indeed, it is likely that several thousand structurally distinct lipid species exist in mammalian cells (see lipidmaps.org).^13, 14^ Together, these lipids have many roles in RCD, as triggers of cell death, as essential components needed for the operation of multi-step RCD pathways, and ultimately, as components of lipid membranes that are physically disrupted (i.e., breached) in various ways during cell death.

The roles of lipids in apoptotic cell death have been studied for some time, and provide a framework for understanding the various roles that lipids can play in non-apoptotic RCD. First, lipids can serve as a signal to initiate apoptosis or transduce an apoptotic signal. Thus, treatment of cells with the saturated fatty acid (SFA) palmitate (16 : 0, annotations herein refer to the number of carbon atoms and points of unsaturation) can trigger apoptosis by causing endoplasmic reticulum (ER) stress,^15^ while ceramide (a sphingolipid; Figure 1a) accumulates in cancer cells exposed to pro-apoptotic signals (e.g., ultraviolet irradiation, the small molecule staurosporine) and has an enigmatic role in transducing this signal, perhaps by damaging intracellular membranes or the plasma membrane.^16, 17, 18^ Second, lipids have important accessory roles in the execution of apoptosis. For example, in the intrinsic apoptosis pathway, oligomerization of the pore-forming BH3 family members BCL2-associated X protein (BAX) and BCL2-antagonist/killer 1 (BAK) on the mitochondrial outer membrane requires the lipids sphingosine-1-phosphate and hexadecenal as specific cofactors.^19^ Furthermore, downstream of BAX and BAK insertion into the mitochondrial outer membrane, oxidization of PUFA-rich mitochondrial cardiolipins (a class of glycerophospholipids synthesized in the mitochondrion) on the outer leaflet of the mitochondrial inner membrane promotes the release of cytochrome C and other key apoptotic effectors from the mitochondria into the cytosol.^20, 21^ Third, lipid-containing membranes are key targets for modification and destruction during apoptosis. As noted above, mitochondrial outer membrane permeabilization is an essential step during the intrinsic apoptotic cascade,^22^ while caspase-mediated cleavage of lipid flippases and scramblases at the plasma membrane leads to enrichment of PS on the outer leaflet of the plasma membrane, a signal essential for the recognition and phagocytosis of apoptotic cells.^23, 24, 25, 26, 27^ These examples highlight the multitude of important roles played by lipids and lipid metabolism in apoptotic RCD.

What roles do lipids play in non-apoptotic RCD? Recent studies suggest important roles for lipids and lipid metabolism in both triggering and executing non-apoptotic RCD. These roles are distinct from those observed during apoptosis but involve similar themes, including a role for certain lipids as triggers of cell death and the centrality of membrane lipid damage to the final lethal process. In this review, we highlight a selection of these emerging links between lipids and non-apoptotic RCD in mammalian cells. We also highlight several areas where our knowledge of the connection between lipids and non-apoptotic RCD is wanting and where new technologies may be useful in studying the roles of lipids in RCD.

## Triggering of non-apoptotic cell death by FAs

FAs are simple lipids composed of elongated hydrocarbon chains with a terminal carboxylic acid (Figure 1a). FAs can be synthesized *de novo* (conjugated to coenzyme A (CoA) as an acyl-CoA), liberated from existing lipids, such as glycerolipids and glycerophospholipids, by lipases (e.g., phospholipases), or taken up by the cell from extracellular sources. In *de novo* FA synthesis, the rate-limiting step is the conversion of acetyl-CoA to malonyl-CoA, the essential unit of FA elongation, by acetyl-CoA carboxylases 1 and 2 (ACC1 and ACC2). Fatty acid synthase (FASN) utilizes malonyl-CoA, acetyl-CoA, and NADPH to synthesize the saturated acyl-CoA palmitoyl-CoA (16 : 0). Palmitoyl-CoA can be further elongated by ER-resident enzymes and desaturated by stearoyl-CoA desaturases (SCDs), for example converting stearoyl-CoA (18 : 0) to oleoyl-CoA (18:1n-9, where n-*x* refers to the position of the first carbon-carbon double bond counting from the methyl end of the carbon chain). Acyl-CoAs can then be incorporated into existing lipids by a large family of acyl transferase enzymes. Acyl-CoAs can also be catabolized in mitochondria and peroxisomes through *β*-oxidation. Mitochondrial *β*-oxidation requires carnitine palmitoyltransferase 1 (CPT1)-mediated fatty acyl-CoA uptake into the mitochondria. Subsequent catabolism produces acetyl-CoA, FADH_2_, and NADH, which can fuel the tricarboxylic acid cycle and ATP synthesis. Inhibition of *de novo* FA synthesis and catabolism is thought to trigger apoptosis, at least in cancer cells, where this has been studied most extensively.^28^ Roles for these processes in non-apoptotic RCD are also now emerging.

### Oleic acid as a trigger for cell death in mammary epithelial cells

**Figure 2:**
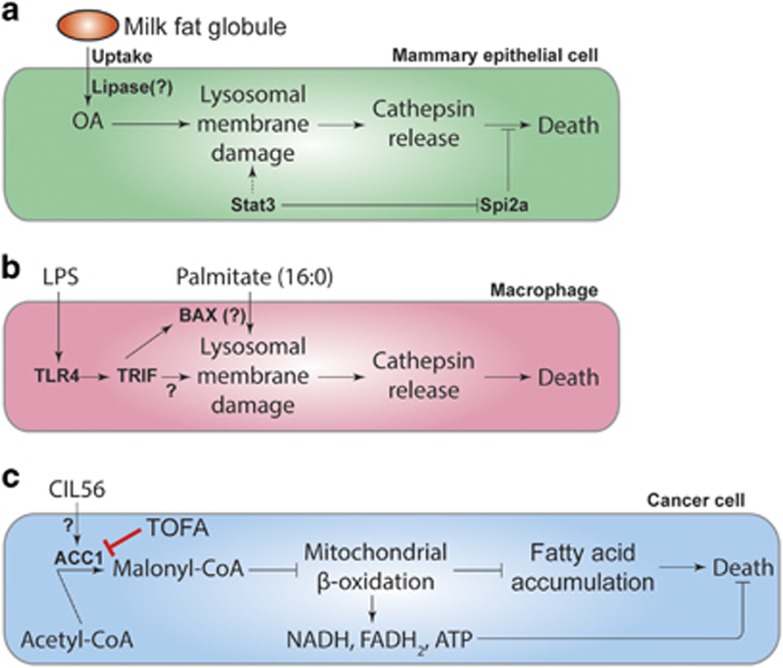
Lipids as triggers of non-apoptotic cell death. (**a**) During the process of post-lactational regression, mammary epithelial cells take up MFGs, a type of storage lipid. MFGs containing OA (18:1n-9) can damage lysosomal membranes, leading to the release of cathepsins and induction of non-apoptotic cell death. It is unclear whether lysosomal damage is triggered by OA conjugated to the glycerol backbone or OA liberated from the glycerol backbone by a lysosomal lipase. Stat3 promotes this process in several ways, including enhancing the size (and potentially the sensitivity to damage) of the lysosomal membrane, upregulating the expression of cathepsins, and inhibiting the expression of the cathepsin inhibitor Spi2a. (**b**) In macrophages, LPS together with palmitate triggers lysosomal damage and non-apoptotic cell death. (**c**) A synthetic small molecule, CIL56, can trigger caspase-independent cell death that is suppressed by deleting ACACA, which encodes ACC1, or inhibiting ACC activity using TOFA. The lethal mechanism is unclear, but may involve the accumulation of malonyl-CoA and inhibition of mitochondrial *β*-oxidation, leading to the simultaneous accumulation of multiple FAs to toxic levels and/or depletion of the products of *β*-oxidation (NADH, FADH_2_, ATP)

An intriguing connection is emerging between the monounsaturated fatty acid (MUFA) oleic acid (OA), lysosomal membrane permeability (LMP) and non-apoptotic RCD (Figure 2a). Lysosomes are intracellular organelles with an enigmatic connection to cell death, being linked to both apoptotic and non-apoptotic cell death in different cells and contexts.^29, 30^ However, following pregnancy and weaning, a lysosome-mediated, non-apoptotic pathway is activated to return the milk-producing mammary epithelium to its pre-pregnancy state (i.e., post-lactational regression, also called involution).^31, 32^ The transcription factor Stat3 is a key regulator of involution that promotes the physical expansion of the lysosome, upregulation of the lysosomal-resident protease cathepsins B and L, and downregulation of serine protease inhibitor 2a (Spi2a), an endogenous serpin-family cathepsin inhibitor. Stat3 also controls a switch from a secretory state (during lactation) to a phagocytic state (during involution). In the phagocytic state, mammary cells phagocytize milk fat globules (MFGs) that contain high levels of OA-containing triacylglycerides. OA liberated from triacylglycerides in the lysosome triggers permeabilization of lysosomes and leakage of intralysosomal cathepsins B and L into the cytosol where they initiate caspase-independent cell death. How OA released from MFGs triggers lysosomal cathepsin leakage is not clear, but insertion of the OA chain, which is ‘kinked' by a single point of unsaturation, into the lysosomal membrane may physically disrupt its integrity and help to open pores through which cathepsins can exit. How cathepsin release causes non-apoptotic cell death remains to be characterized.

### Palmitate as a trigger for cell death in macrophages

A different interaction between lipids and the lysosome can trigger non-apoptotic RCD in macrophages. When these cells are exposed to palmitate in combination with lipopolysaccharide (LPS, a lipid-modified sugar found on the surface of Gram-negative bacteria), cells undergo an unusual form of non-apoptotic cell death that is not blocked by broad-spectrum caspase inhibitors or the necroptosis inhibitor necrostatin-1 (Nec-1; Figure 2b).^33, 34^ This pathway requires the LPS receptor Toll-like receptor 4 (TLR4) and the downstream component TIR-domain-containing adapter-inducing interferon-*β* (TRIF), which primes lysosomes in an unknown way for palmitate-induced damage and cathepsin release. In the absence of LPS treatment, palmitate causes only minimal levels of cell death in these cells, and the link between palmitate, the TLR4-TRIF pathway, lysosomes, and cell death is obscure. It is known that palmitate, in complex with BAX, can induce LMP;^35^ it is therefore possible that TLR4-TRIF signaling could enhance BAX expression or deplete the cell of an endogenous BAX inhibitor, allowing a palmitate–BAX complex to cause lysosomal damage.

### Generalized FA accumulation as a trigger for non-apoptotic cell death

Recently, an unusual example of how lipid accumulation can trigger non-apoptotic RCD has emerged from the study of a synthetic small molecule named CIL56 (Figure 2c). Treatment of human HT-1080 fibrosarcoma cells with CIL56 causes the accumulation of numerous SFAs, MUFAs, and PUFAs, and results in caspase-independent RCD.^36^ Genetic screening identified *ACACA* (encoding ACC1) as a gene essential for CIL56-induced cell death.^36^ Moreover, a small molecule ACC inhibitor, 5-(tetradecyloxy)-2-furoic acid (TOFA), prevents both CIL56-induced cell death and the observed changes in SFA, MUFA, and PUFA levels within the cell.^36^ One model is that CIL56 stimulates ACC activity, leading to the accumulation of malonyl-CoA. Malonyl-CoA is an endogenous negative regulator of CPT1^28^ and inhibition of CPT1-dependent mitochondrial FA *β*-oxidation activity could account for the accumulation of multiple FA species in CIL56-treated cells. The accumulation of one or more FAs may be directly toxic itself, through direct effects on membrane-enclosed organelles or the plasma membrane. Alternatively or in parallel, the inhibition of mitochondrial *β*-oxidation may lead to the depletion of metabolites needed for cell survival (e.g., ATP).^37^ Although the precise target and mechanism of action of this lethal compound remains to be resolved, these results suggest that small molecule-mediated perturbations of FA metabolism can cause non-apoptotic RCD.

## Essential roles for lipids in the execution of non-apoptotic cell death

The examples presented above suggest that FAs can in some contexts trigger non-apoptotic RCD. In this section, we consider examples where lipids are required for the execution of non-apoptotic RCD by acting as signaling molecules, modulators of lethal pathways, or essential nodes in the lethal pathways themselves.

### Necroptosis

**Figure 3:**
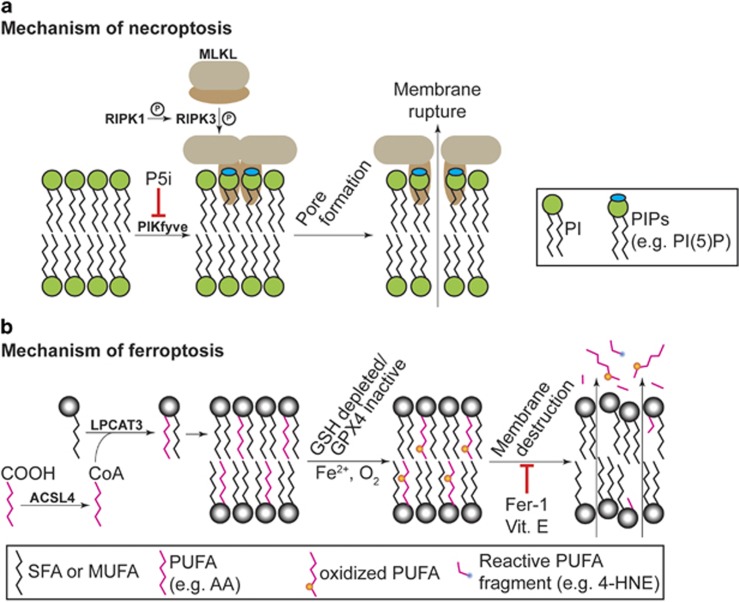
Role for lipids as executioners of non-apoptotic cell death. (**a**) Necroptosis involves the activation of RIPK1, which phosphorylates RIPK3 (denoted by the circled P), which then in turn phosphorylates MLKL. MLKL interacts with specific PIPs on lipid bilayers within the cell, including PI(5)P and PI(4,5)P_2_. The formation of PIPs from PIs requires various PI kinases such as PIKfyve. Phosphorylated MLKL undergoes a confirmational change that allows it to bind PIPs on the plasma membrane and, presumably, form a lethal membrane pore. The stoichiomety of the pore-forming MLKL oligomer is debated. (**b**) Ferroptosis requires membrane-resident PUFAs, depicted as pink chains, such as arachidonic acid (AA, 20:4n-6). For death to proceed, PUFAs must be acylated by acyl-CoA synthetase long-chain family member 4 (ACSL4) and inserted into lysophospholipids by lysophosphatidylcholine acyltransferase 3 (LPCAT3). Ferroptosis involves oxidation (orange dots) of membrane PUFAs. It is thought that this leads to fragmentation of these oxidized species, generating toxic aldehydes like 4-hydroxynonenal (4-HNE). Cell death is due to physical destruction of the membrane. Death can be prevented by synthetic or natural lipophilic antioxidants such as ferrostatin-1 (Fer-1) or vitamin E (Vit. E), respectively.

Necroptosis is a non-apoptotic form of RCD that is implicated in homeostatic and pathological cell death in the immune system, brain, and other tissues.^38^ Necroptosis can be induced by cytokines such as tumor necrosis factor-alpha (TNF-*α*). In the presence of caspase inhibitors, TNF-*α* induces the formation of a multiprotein complex, called the necrosome, that promotes necroptosis.^4^ In this pathway, receptor-interacting serine/threonine-protein kinases 1 and 3 (RIPK1 and RIPK3) heterodimerize, leading to the recruitment of MLKL and its subsequent phosphorylation on T387 and S358 (in the human protein) by RIPK3.^39^ Once phosphorylated, MLKL translocates to the plasma membrane (as well as intracellular membranes), oligomerizes, and causes cell death (Figure 3a).^40, 41, 42, 43^ Lipids are essential for this terminal process: charged amino acids in the N-terminal coiled-coil domain/4-helical bundle of phosphorylated MLKL bind specifically to membrane PIPs (Figure 1b).^41, 42, 43^ Based on studies performed using liposomes, MLKL oligomerization at the membrane is necessary and sufficient to cause membrane leakage, suggesting that *in vivo* MLKL oligomers may form a pore in the plasma membrane to cause the release of intracellular contents, loss of ionic homeostasis, and cellular rupture.^41^ PIPs, including PI(5)P and PI(4,5)P_2_, are required for MLKL membrane targeting, as liposomes containing only PIs, but not PIPs, do not exhibit MLKL-dependent leakage.^41^ Interestingly, specific MLKL mutants can be used to dissociate membrane binding from membrane permeabilization, suggesting that these processes are independent.^43^ Imaging studies in human cells reveal phosphorylated MLKL in discrete puncta, possibly indicating that membrane permeabilization is initiated at specific sites on the plasma membrane.^41^ Small molecule inhibitors that putatively block the formation of PI(5)P by phosphoinositide kinase, FYVE finger containing (PIKfyve) protein, or the formation of PI(4,5)P_2_ from PI(3,4,5)P_3_ by phosphatase and tensin homolog (PTEN), partially suppress and delay TNF-*α*-induced necroptosis in mouse L929 cells,^42^ indicating that PI(5)P and PI(4,5)P_2_ may be the most potent MLKL-binding lipids. These results highlight lipid composition and phosphorylation status of specific membrane glycerophospholipids as key mediators of necroptosis.

### Pyroptosis

Pyroptosis is a highly inflammatory form of non-apoptotic RCD triggered in immune cells in response to various pathogen-associated molecular patterns (PAMPs) and damage-associated molecular patterns (DAMPs), including bacterial proteins, cholesterol crystals, silica, asbestos, and extracellular ATP.^44^ PAMPs and DAMPs trigger the formation of multiprotein ‘inflammasome' complexes that activate caspase-1 or caspase-4/5 (caspase-11 in mouse),^45^ which in turn can promote the formation of membrane pores, loss of ionic balance, cell rupture, and release of intracellular contents, including inflammatory cytokines such as interleukin-1*β*.^46^ Gasdermin D was recently identified as a caspase-1/4/5/11 substrate that is essential for pyroptosis.^47, 48^ The cleaved N-terminal domain of gasdermin D is sufficient to trigger death, and one possibility is that this fragment acts at the membrane in a manner analogous to MLKL in necroptosis to form a membrane pore itself or to cause the oligomerization of other pore-forming proteins. If so, one prediction is that the interaction of the N-terminal gasdermin D domain with membrane lipids would be essential for membrane permeation.

Although a role for lipids in the execution of pyroptosis remains speculative, stronger evidence exists to suggest a role for specific lipids as modulators of this process. For example, in mouse macrophages primed to undergo pyroptosis, inhibition of FASN-mediated *de novo* FA synthesis using the small molecule inhibitors cerulenin and C75 attenuates the assembly of the nucleotide-binding oligomerization domain (NOD)-like receptor 3 (NLRP3) inflammasome and caspase-1 activation.^49^ Conversely, exposure to exogenous palmitate can enhance NLRP3-dependent caspase-1 activation.^50^ Mechanistically, palmitate may promote inflammasome assembly and caspase-1 activation indirectly, by serving as a substrate for CPT1-dependent mitochondrial *β*-oxidation, a process that leads to enhanced reactive oxygen species (ROS) production (a known inducer of inflammasomes).^50, 51^ If this mechanism is important, then any change in intracellular FA metabolism that enhances *β*-oxidation may serve to promote inflammasome activation and cell death, while conditions that suppress FA production or catabolism would inhibit this mechanism.

### Ferroptosis

Ferroptosis is an iron-dependent, oxidative form of non-apoptotic RCD that has been implicated in pathological cell death in brain, kidney, and heart tissues.^6, 52, 53, 54, 55, 56^ Ferroptosis can be triggered by the depletion of intracellular glutathione or inhibition of the essential glutathione (GSH)-dependent lipid hydroperoxide detoxifying enzyme GPX4.^57^ Normally, GSH-dependent GPX4 activity reduces potentially toxic lipid peroxides (L-OOH) to non-toxic lipid alcohols (L-OH). The current working model is that once GPX4 is inactivated, L-OOHs accumulate and interact with iron, resulting in iron-catalyzed formation of lipid radicals (L-O^•^) that are lethal to the cell (Figure 3b).^52, 57^ Indeed, depletion of GSH or direct inhibition of GPX4 results in the iron-dependent accumulation of ROS, accumulation of lysophospholipids (i.e., glycerophophospholipids lacking one fatty acyl chain), and depletion of several PUFAs such as arachidonic acid (AA, 20:4n-6).^52, 54, 57^ The accumulation of lysophospholipids and the depletion of specific PUFAs suggest that specific oxidized PUFAs are cleaved from the glycerophospholipid backbones (e.g., possibly by phospholipase A2^54^) and subsequently degraded or destroyed. Strikingly, deletion of *ACSL4* or *LPCAT3*, which encode enzymes required for the reacylation of membrane lysolipids with AA and other PUFAs, prevents ferroptosis induced by GPX4 inactivation (Figure 3b).^36^ Thus, to proceed, ferroptosis requires AA and likely other PUFAs prepositioned in the membrane to serve as targets for O_2_-mediated oxidation and iron-catalyzed free radical production. As would be predicted from this model, cells are protected from ferroptosis by exogenous small molecules that neutralize toxic lipid ROS or block the formation of lipid peroxyl radicals. This includes small molecule lipophilic antioxidants (trolox, butylated hydroxytoluene, ferrostatin-1) and iron chelators (deferoxamine, ciclopirox).^6, 58, 59^ Supplementation with the prenol lipid antioxidant vitamin E (*α*-tocopherol) (Figure 1a) can prevent ferroptosis in cell culture^58^ and the endogenous levels of this lipid also presumably impacts the ability of the cell to undergo ferroptosis following GPX4 inactivation *in vivo* (e.g., Wortmann *et al.*^60^ and Saito *et al.*^61^).

A major question concerns how PUFA oxidation leads to ferroptotic cell death. To date, there is no evidence that ferroptosis involves formation of a protein-based pore, although this possibility cannot currently be excluded. Oxidative destruction of plasma membrane PUFAs may lead to the formation of gaps in the plasma membrane, resulting in loss of ionic homeostasis. Alternatively or in parallel, the accumulation of highly oxidized PUFAs (or derivative fragments thereof, such as 4-hydroxynonenal (4-HNE)) could directly inactivate essential intracellular proteins or trigger additional death-promoting events.^62^ Consistent with this latter possibility, high expression of three enzymes (*AKR1C1*, *AKR1C2*, and *AKR1C3*) that can detoxify reactive aldehydes such as 4-HNE^63^ is associated with resistance to ferroptosis.^62^

A recent report demonstrates that expression of the tumor suppressor protein p53 can modulate the sensitivity to ferroptosis *in vitro* and *in vivo*.^64^ Wild-type p53 can enhance the expression of numerous lipid metabolic enzymes, including CPT1 and acyl-CoA dehydrogenase family member 11 (ACAD11), to promote FA oxidation,^65, 66^ while mutant p53 can upregulate *de novo* FA synthesis genes including *FASN*, *SCD,* and multiple genes in the mevalonate pathway that contribute to the synthesis of cholesterol and various prenol lipids.^67^ While speculative, one possibility is that p53-dependent effects on lipid metabolism could modulate ferroptosis sensitivity by altering lipid metabolism or membrane lipid composition.

## Challenges and open questions

Lipids have important and diverse roles as triggers and executioners of non-apoptotic RCD. There are a number of areas where significant gaps exist in our understanding of the link between lipids and non-apoptotic cell death, which we discuss below. Additionally, new technologies to manipulate, image and quantify lipids should help better understand the roles of specific lipids, lipid metabolic enzymes and lipid metabolic pathways in RCD (Box 1).

### Understanding lethal pathways triggered by lipid accumulation

In most cases, how certain lipids activate specific non-apoptotic RCD pathways is not clear. For example, as noted above, the precise non-apoptotic RCD pathway that is activated by stimuli such as OA release from MFGs in mammary epithelial cells, or exposure to LPS+palmitate in macrophages, remains unclear. Likewise, glycerolipids with a single fatty acyl substituent (lysophosphatidylcholine and lysophosphatidic acid) trigger pyroptosis in human aortic endothelial cells though an unknown molecular mechanism.^68^

In a related vein, some stimuli trigger non-apoptotic death involving recognizable components of a specific pathway, but in an unusual or unexpected way. Thus, in human umbilical endothelial cells, palmitic acid induces a form of necroptosis that is RIPK3 dependent but RIPK1 independent,^69^ implying that this treatment can bypass the need for RIPK1 activation. Conversely, treatment of SH-SY5Y neuroblastoma cells and Jurkat cells with the oxysterol 24(*S*)-hydroxycholesterol (24*S*-OHC) leads to cell death that is RIPK1 dependent, but RIPK3 and MLKL independent.^70, 71, 72^ Further studies of these examples should help determine whether certain lipids engage truly novel lethal pathways or activate known non-apoptotic RCD pathways, but in unusual or cell type-specific ways.

### Understanding context specificity and crosstalk

Depending on the context, the same lipid can have different roles in cell death. As described above, OA triggers lysosome-dependent non-apoptotic RCD in mammary epithelial cells,^32^ but acts to prevent apoptotic RCD in cells treated with high levels of palmitate or when mechanistic target of rapamycin (mTOR) signaling is aberrantly activated.^73, 74^ Palmitate together with LPS induces a predominantly non-apoptotic cell death phenotype in macrophages,^33^ while in pancreatic cells, palmitate triggers apoptosis.^75^ Understanding how the same lipid can have opposing roles in different cell types is an important unsolved problem.

Disruption of lipid homeostasis may also connect two different lethal processes. In mouse erythroid precursor cells inactivation of GPX4, a canonical trigger for ferroptosis,^57^ leads to the accumulation of toxic lipid intermediates that covalently modify caspase-8 and trigger necroptosis in the absence of death receptor stimulation.^76^ This suggests the existence of lipid-mediated crosstalk between the ferroptosis and necroptosis pathways. This link is likely cell-type specific, as canonical inhibitors of necroptosis do not block ferroptosis in a variety of cell lines^6, 54^ and mouse embryonic fibroblasts lacking *Ripk1*, or mouse L929 fibrosarcoma cells where *Ripk3* is depleted, are fully competent to undergo ferroptosis.^54^ In a related example, GPX4 inhibition can sensitize cancer cells to apoptosis induced by second mitochondrial-derived activator of caspases (SMAC) mimetics,^77^ connecting ferroptosis to apoptotic cell death pathways. Although more work is required to define these links, given the possibility that cells *in vivo* may be exposed to more than one lethal stimulus simultaneously, it will be important to investigate whether two lethal pathways can operate in parallel, whether lethal pathways are organized in some kind of hierarchy or whether activation of multiple pathways simultaneously generate new hybrid lethal pathways.

### Understanding the role of lipid membrane repair

Membrane breaching occurs in all forms of RCD. In at least some cases, death-promoting stimuli activate both lethal and protective responses targeting the membrane. For example, bacterial pore-forming toxins (PFTs) such as aerolysin or *Staphylococcus α*-toxin cause K^+^ efflux from the cell, which ultimately leads to inflammasome-dependent caspase-1-dependent processing and nuclear translocation of the cholesterol and lipid biosynthetic master regulators sterol-regulatory element-binding proteins 1 and 2 (SREBP1/2).^78^ SREBP1/2 upregulates transcription of *FASN* and *HMGCR* (encoding 3-hydroxy-3-methylglutaryl-CoA reductase, the rate-limiting enzyme required for cholesterol synthesis), resulting in the accumulation of cholesterol and other lipids.^78^ When SREBP1/2 processing is inhibited, cells are hypersensitized to PFT-induced cell death.^78^ Although it is not known how cholesterol and other lipids help the cell resist death, a plausible mechanism is that newly synthesized lipids are used to help stabilize or repair damaged membranes. Such a response would help ensure that cell death only proceeds in cells receiving a strong lethal stimulus, allowing damage due to ‘accidental' exposures to be repaired.

Ultimately, to ensure cell death, processes that promote the stability and function of the membrane must be overcome. Different forms of acute membrane damage associated with non-apoptotic RCD, from mechanical damage to the insertion of bacterial PFTs into the membrane, are repaired by spontaneous membrane resealing for small diameter lesions (<2 nm) or Ca^2+^ influx-triggered repair for larger injuries.^79^ Ca^2+^-dependent repair involves a number of membrane-linked changes, including fusion with intracellular vesicles and organelles (e.g., lysosomes), endocytosis, protrusion or ‘blebbing' of the membrane and exocytosis.^79, 80, 81^ These responses occur within seconds to minutes of membrane damage and represent the immediate attempt to prevent widespread loss of membrane homeostasis and eject damaged portions of the membrane (such as those containing bacterial PFTs or oxidatively damaged lipids) from the cell surface. One intriguing possibility is that non-apoptotic pathways subvert these immediate responses to membrane damage, thereby promoting death.

### Understanding how modulation of lipid metabolism by other metabolic processes impacts cell death

An important goal is to better understand the links between non-apoptotic RCD and specific lipid metabolic pathways and processes.^82^ The execution of ferroptosis, in particular, requires the uptake of glutamine and the production of citrate, processes that may support high rates of *de novo* lipid synthesis.^6, 55, 83^ More broadly, lipid storage organelles such as lipid droplets (LDs) and key lipid-modifying pathways such as autophagy may have roles as modulators of non-apoptotic RCD. LDs are dynamic structures that comprise a single membrane enclosing a core containing mostly neutral lipids (e.g., triacylglycerides, Figure 1a) and cholesterol esters. Apoptosis-inducing agents, such as etoposide, inhibit mitochondrial *β*-oxidation and promote FA incorporation in LDs.^84^ Whether these organelles are linked to non-apoptotic RCD is unclear. However, in one example cited above, the treatment of SH-SY5Y neuroblastoma cells and Jurkat cells with 24*S*-OHC leads to the acyl-CoA:cholesterol acyltransferase 1 (ACAT1)-dependent formation of LDs and toxic accumulation of esterified 24*S*-OHC derivatives, presumably in LDs.^70, 71, 72^

Autophagy is a process of cellular ‘self-digestion' that involves the formation of double-membrane intracellular vesicles that can fuse with lysosomes to degrade intracellular contents (i.e., protein and lipids). While controversial, in mammalian cells autophagy is generally thought to be protective rather than lethal.^85, 86^ However, in human umbilical endothelial cells, treatment with palmitic acid is lethal and cell death is partially suppressed by inhibition of the autophagy genes *VPS34* and *ATG7*.^69^ This suggests that autophagy may promote death in this context. Conversely, since the process of autophagy can consume lipids (i.e., lipophagy),^87^ it is intriguing to speculate that this process could prevent non-apoptotic RCD. For example, lipophagy could help maintain ATP levels under conditions that would normally lead to ATP depletion and necrotic cell death.^88^ Another possibility is that high levels of lipophagy could block non-apoptotic RCD by consuming key lipids or FAs that would otherwise trigger cell death.

### Understanding cell non-autonomous effects

In addition to cell-autonomous roles in non-apoptotic RCD, lipids also have systemic roles in mediating cell death *in vivo*. For example, treatment of peritoneal macrophages with a chimeric toxin activates the NAIP5/NLRC4 inflammasome and leads to an eicosanoid storm, independent of cytokine production, that is acutely lethal to mice.^89^ Eicosanoids are paracrine signaling molecules, including prostaglandins and leukotrienes, derived from the oxidation of AA by the cyclooxygenase and lipoxygenase pathways.^90, 91^ Production of these molecules is highly upregulated during infection and eicosanoids act as both pro- and anti-inflammatory non-cell autonomous signals.^92^ During ferroptosis, oxidized derivatives of AA, including 5-hydroxyeicosatetraenoic acid (5-HETE), 11-HETE, and 15-HETE, are released into the surrounding medium of dying cells.^54^ If ferroptosis also occurs in the body, then these molecules could likewise have important signaling roles, such as attracting immune cells to sites of tissue damage.^93^ In this manner, the release of oxidized lipid derivatives could have an analogous role to the externalization of PS during apoptosis, facilitating non-cell autonomous processes essential for the proper disposal of dead and dying cells.

### Understanding the causes of pathological cell death

A number of inherited genetic disorders, including Niemann-Pick disease, cerebrotendinous xanthomatosis, hereditary spastic paraparesis type 5, and a spectrum of peroxisomal *β*-oxidation disorders, are caused by mutations in specific lipid metabolic genes. These inborn errors of metabolism (IEMs) result in the accumulation of particular lipids and cell type-specific cell death, typically in the nervous system (Table 1).^94, 95, 96, 97, 98, 99, 100, 101^ How lipid accumulation (or depletion) triggers death in these cells, and whether this involves apoptotic or non-apoptotic pathways, is generally not well understood. Additional studies of these disorders could unveil new mechanisms of lipid-triggered or lipid-mediated non-apoptotic RCD. For example, in a mouse model of Niemann-Pick disease Type C, a disease characterized by the aberrant accumulation of cholesterol and other lipids in endosomes and lysosomes due to mutations in *Npc1*, neuronal cell death is not prevented by transgenic expression of the anti-apoptotic protein Bcl-2, suggesting that death could involve activation of a non-apoptotic pathway.^102^ Failure to repair oxidatively damaged lipids, due to mutations that impair the function of the GSH-GPX4 pathway, is another example of how disruption of normal lipid metabolism can impair organismal function^61^ and further study of these disorders could lead to additional insights into the regulation of ferroptotic cell death *in vivo*.

### Treating disease

It may be possible to treat disease by modulating (enhancing or suppressing) specific non-apoptotic RCD pathways. Targeting the lipid-dependent aspects of these processes may provide novel routes to do so. For example, certain lung cancer cells can be induced to die via necroptosis by the sphingosine analog drug FTY720 (fingolimod) through a mechanism involving protein phosphatase 2A-dependent activation of RIPK1.^103^ One interesting prediction consistent with this result is that FTY720 might mimic the function of an endogenous lipid that normally promotes necroptosis to limit tumor formation. Drug repurposing may also open avenues to the manipulation of lipid-dependent non-apoptotic RCD in humans. Two reports have suggested that it may be possible to induce ferroptosis, at least in some cells, using the multikinase inhibitor sorafenib and the antineoplastic agent altretamine.^62, 104^ Although sorafenib appears to trigger ferroptosis by blocking GSH synthesis, altretamine can directly inhibit GPX4.^62, 104^ It remains to be seen whether evidence of ferroptosis can be found in patients receiving these drugs.

In addition to inducing cell death through modulation of lipid metabolism, inhibiting lipid-dependent processes may be useful in some contexts. For example, synthetic lipophilic antioxidants that inhibit ferroptosis have been shown to block pathological cell death in brain and kidney tissues.^52, 53^ A better understanding of how lipids and lipid metabolism impact non-apoptotic RCD should allow for the development of improved therapies targeting these processes in the future. Quarato *et al* (Molecular Cell, Feb 18;61(4): 589-601) report that membrane PI(4,5)P2 is the preferred binding partner for MLKL in cells undergoing necroptosis.

## Figures and Tables

**Table 1:** Examples of inherited disorders of lipid metabolism linked to cell death

**Disease**	**OMIM identifier**	**Gene**	**Lipid-dependent death phenotype**
Niemann-Pick disease (Types A, B)	257200, 607616	*SMPD1*	Failure to catabolize sphingomyelin to ceramide in the lysosome. Leads to neurodegeneration with death typically by age 3 (in Type A) and liver disease in adulthood (in Type B)
Niemann-Pick disease (Type C)	257220	*NPC1*, *NPC2*	Accumulation of cholesterol and glycerosphingolipids in endosomes and lysosomes, leading to pathological cell death
Hereditary spastic paraparesis type 5	270800	*CYP7B1*	Defects in cholesterol metabolism, particularly the formation of hydroxysteroids, with a corresponding accumulation of oxysteroid precursors (25-hydroxycholesterol) and degeneration of motor neurons
Cerebrotendinous xanthomatosis	213700	*CYP27A1*	Defects in cholesterol metabolism leading to accumulation of toxic cholesterol deposits in the cerebellum and other tissues
Refsum disease	266500	*PHYH*	Failure to metabolize phytanic acid (a bacterially-produced branched chain lipid) in the peroxisome, leading to neuronal cell death
Peroxisome biogenesis disorder 1A (Zellweger)	214100	*PEX1*	Defects in peroxisome biogenesis leading to the accumulation of very long chain fatty acids (VLCFAs, e.g., C26:0), neuronal cell death and death before 1 year of age in severe cases
Adrenoleukodystrophy	300100	*ABCD1*	Defect in peroxisomal VLCFA metabolism leading to toxic VLCFA accumulation, neuronal demyelination and death
Krabbe disease	245200	*GALC*	Defect in the catabolism of galactocerebrosides (sphingolipids conjugated to galactose) leading to the accumulation of specific sphingolipids (e.g., psychosine), destruction of oligodendrocytes in the nervous system and death by the age of two in severe cases

Inherited mutations in specific lipid metabolic genes can lead to the accumulation of particular lipids and pathological cell death. Eight examples are shown. In most cases, the type of cell death triggered by abnormal lipid accumulation is unclear. Further information can be found at the OMIM website (www.omim.org/).

## New tools and technologies for analyzing the role of lipids in cell death

Compared with proteins and nucleic acids, the tools to investigate and manipulate lipids in the cell are historically less accurate, less sophisticated and less accessible. However, new tools and technologies are emerging that should help study the roles of lipids and lipid metabolism in RCD. Here, we provide four examples of how specific technologies could be used. (**a**) Pooled CRISPR/Cas9 genetic screening technology^105^ could be used to search for new modulators (enhancers or suppressors) of lipid-triggered non-apoptotic RCD (e.g., Condition A=control and condition B=lipid stimulation). (**b**) Computational reconstruction and modeling of metabolic networks in mammalian cells (e.g., using Recon 2, humanmetabolism.org)^106^ could be used to predict the metabolic consequences of exogenous lipid addition or the effects of enzyme inactivation on the levels of various endogenous lipids in the cell. (**c**) The localization and fate of specific lipids or lipid-rich organelles (e.g., lipid droplets, LDs) in live *versus* dying cells could be interrogated kinetically in live cells using methods such as linear Raman imaging spectroscopy,^107^ coherent anti-Stokes Raman scattering microscopy, and stimulated Raman scattering (SRS) microscopy.^108^ For example, cells fed an alkyne-labeled fatty acid (17-octadececynoic acid) can incorporate this molecule into LDs and membrane phospholipids (PLs). The abundance and localization of labeled LDs and PLs can then be monitored inside the cell using SRS.^108^ (**d**) Advances in mass spectrometry-based lipidomics^109, 110, 111, 112^ are enabling the identification of individual lipid species whose levels are perturbed in dying *versus* live cells.^36, 52, 57^ A related approach to monitor the relative abundance of different fatty acids (FAs) between samples involves post-lysis labeling of FAs with unique stable isotope tags (free fatty acid stable isotope tagging (FFAST)) whose mass differences can be resolved by LC-MS/MS.^111^ This advance could help improve the precision of comparative lipidomics comparing live and dying cells.

## Abbreviations

RCD: regulated cell death
MLKL: mixed lineage kinase domain-like
PUFA: polyunsaturated fatty acid
GPX4: glutathione peroxidase 4
FAs: fatty acids
PC: phosphatidylcholine
PI: phosphatidylinositol
PS: phosphatidylserine
PIPs: phosphatidylinositol phosphates
SFA: saturated fatty acid
MUFA: monounsaturated fatty acid
LA: linoleic acid
ER: endoplasmic reticulum
BAX: BCL2-associated X protein
BAK: BCL2-antagonist/killer 1
CoA: coenzyme A
ACC1: acetyl-CoA carboxylase 1
ACC2: acetyl-CoA carboxylase 2
FASN: fatty acid synthase
SCD: stearoyl-CoA desaturase
CPT1: carnitine palmitoyltransferase 1
OA: oleic acid
LMP: lysosomal membrane permeability
Spi2a: serine protease inhibitor 2a
MFGs: milk fat globules
LPS: lipopolysaccharide
Nec-1: necrostatin-1
TLR4: Toll-like receptor
TRIF: TIR-domain-containing adapter-inducing interferon-*β*
TOFA: 5-(tetradecyloxy)-2-furoic acid
TNF-*α*: tumor necrosis factor-alpha
RIPK1: receptor-interacting serine/threonine-protein kinase 1
RIPK3: receptor-interacting serine/threonine-protein kinase 3
PIKfyve: phosphoinositide kinase
FYVE: finger containing
PTEN: phosphatase and tensin homolog
PAMPs: pathogen-associated molecular patterns
DAMPs: damage-associated molecular patterns
NLRP3: nucleotide-binding oligomerization domain-like receptor 3
ROS: reactive oxygen species
GSH: glutathione
L-OOH: lipid peroxide
L-OH: lipid alcohol
L-O•: lipid radical
AA: arachidonic acid
ACSL4: acyl-CoA synthetase long-chain family member 4
LPCAT3: lysophosphatidylcholine acyltransferase 3
Fer-1: ferrostatin-1
Vit. E: vitamin E
4-HNE: 4-hydroxynonenal
ACAD11: acyl-CoA dehydrogenase family member 11
SRS: stimulated Raman scattering
PLs: phospholipids
FFAST: free fatty acid stable isotope tagging
LC-MS/MS: liquid chromatography-tandem mass spectrometry
24*S*-OHC: 24(*S*)-hydroxycholesterol
mTOR: mechanistic target of rapamycin
SMAC: second mitochondrial-derived activator of caspases
PFTs: pore-forming toxins
SREBP1/2: sterol-regulatory element-binding proteins 1 and 2
HMGCR: 3-hydroxy-3-methylglutaryl-CoA reductase
LDs: lipid droplets
ACAT1: acyl-CoA:cholesterol acyltransferase 1
HETE: hydroxyeicosatetraenoic acid
IEMs: inborn errors of metabolism
